# Improving ART programme retention and viral suppression are key to maximising impact of treatment as prevention – a modelling study

**DOI:** 10.1186/s12879-017-2664-6

**Published:** 2017-08-09

**Authors:** Nicky McCreesh, Ioannis Andrianakis, Rebecca N. Nsubuga, Mark Strong, Ian Vernon, Trevelyan J. McKinley, Jeremy E. Oakley, Michael Goldstein, Richard Hayes, Richard G. White

**Affiliations:** 1 London School of Hygiene and Tropical Medicine, Keppel Street, London, WC1E 7HT UK; 2 MRC/UVRI Research Unit on AIDS, P.O. Box 49, Entebbe, Uganda; 3 School of Health and Related Research, The University of Sheffield, 30 Regent Street, Sheffield, S1 4DA UK; 4 Department of Mathematical Sciences, Durham University, Lower Mountjoy, Stockton Road, Durham, DH1 3LE UK; 5 College of Engineering, Mathematics and Physical Sciences, University of Exeter, Penryn Campus, Penryn, TR10 9FE UK; 6 School of Mathematics and Statistics, University of Sheffield, The Hicks Building, Hounsfield Road, Sheffield, S3 7RH UK

**Keywords:** HIV, ART, Uganda, Transmission, Sub-Saharan Africa, Retention

## Abstract

**Background:**

UNAIDS calls for fewer than 500,000 new HIV infections/year by 2020, with treatment-as-prevention being a key part of their strategy for achieving the target. A better understanding of the contribution to transmission of people at different stages of the care pathway can help focus intervention services at populations where they may have the greatest effect. We investigate this using Uganda as a case study.

**Methods:**

An individual-based HIV/ART model was fitted using history matching. 100 model fits were generated to account for uncertainties in sexual behaviour, HIV epidemiology, and ART coverage up to 2015 in Uganda. A number of different ART scale-up intervention scenarios were simulated between 2016 and 2030. The incidence and proportion of transmission over time from people with primary infection, post-primary ART-naïve infection, and people currently or previously on ART was calculated.

**Results:**

In all scenarios, the proportion of transmission by ART-naïve people decreases, from 70% (61%–79%) in 2015 to between 23% (15%–40%) and 47% (35%–61%) in 2030. The proportion of transmission by people on ART increases from 7.8% (3.5%–13%) to between 14% (7.0%–24%) and 38% (21%–55%). The proportion of transmission by ART dropouts increases from 22% (15%–33%) to between 31% (23%–43%) and 56% (43%–70%).

**Conclusions:**

People who are currently or previously on ART are likely to play an increasingly large role in transmission as ART coverage increases in Uganda. Improving retention on ART, and ensuring that people on ART remain virally suppressed, will be key in reducing HIV incidence in Uganda.

## Background

The Joint United Nations Programme on HIV/AIDS (UNAIDS) ‘fast-track targets’ call for fewer than 500,000 new infections in adults per year by 2020 [1], down from an estimated 2.1 million in 2015 [2]. 65% of new infections in 2015 occurred in sub-Saharan Africa, and 83,000 of these new infections occurred in Uganda, with only South Africa, Nigeria, and India having larger numbers [2]. Uganda had an adult (15–49 years) prevalence of HIV of 7.3% at the time of the last national prevalence survey in 2011, with an estimated 39% of HIV-infected adults receiving ART in 2013 [3].

We currently have a limited understanding of who is transmitting HIV, and how this varies by setting. A range of studies have attempted to estimate the proportion of transmission that occurs during primary infection, but a large amount of uncertainty remains [4], and it is likely that the proportion varies between different settings. We have even less understanding of the contribution to overall transmission of people at different stages of the anti-retroviral therapy (ART) care pathway, and how this will change as treatment coverage increases [5, 6]. While ART greatly reduces transmission risk [7], transmission has been observed to occur from people receiving ART, albeit at a low rate, and it is plausible that the rate of transmission will be higher among people not part of research cohorts [7]. An intriguing molecular study conducted in Switzerland [8] suggests that, in that setting, nearly half of transmission by people with post-primary infections may occur after they have first initiated ART, with much occurring during treatment interruptions. The approach used in the study can only give very rough estimates however, due to the difficulties in determining the exact date of transmission. In addition, it can only be used in countries that have routine viral sequencing.

Treatment-as-prevention is a key component of the UNAIDS strategy to reduce HIV incidence. To meet the ambitious goals of the strategy, it is necessary to develop a better understanding of the relative contribution to overall HIV transmission of people at different stages of the care pathway. This will assist policy makers to focus intervention services at populations where they are likely to have the greatest effect. We investigate this using a mathematical model of HIV transmission and ART scale-up, using Uganda as a case study.

## Methods

### Model structure

A dynamic, individual-based model of HIV transmission and ART scale-up was developed in NetLogo [9]. The model simulates births, deaths, and population growth; the formation and dissolution of sexual partnerships; HIV transmission; pre-ART care and first and second line ART; and the development and transmission of drug resistance. The model was designed to accurately represent the key features of major routes into and through pre-ART care and ART in Uganda, as well as attrition and re-entry at different stages. A full description of the model structure is given in McCreesh et al. [10].

### Data, model parameterisation and fitting

The model was fitted to data on the estimated adult (15–49 year-old) male and female population size in Uganda in 2015, and the growth in population between 1950 and 2015. As no detailed, representative data on sexual behaviour were available from Uganda as a whole, the model was fitted to data on sexual behaviour from a rural open general population cohort in South-West Uganda [11–13]. This included data on the prevalence and incidence of sexual partnerships, and the prevalence of partnership concurrency. Two sexual behaviour risk groups (high and low partnership incidence) and two concurrency groups (high and low concurrency) were simulated. All partnerships in the model had the same duration.

The model was fitted to data from UNAIDS surveys on the overall HIV prevalence in adults in Uganda in 1991, and the male and female adult HIV prevalences in 2004 and 2011 [3]. Twenty-seven fitted outputs were used to ensure that the model accurately represented HIV care and ART scale-up in Uganda. These included, in a number of different years, the proportion of men and women ever tested for HIV, the proportion of HIV positive people on ART, the proportion of people in HIV care on ART, the proportion of people starting ART with a CD4 count <250 cells/μl (cut-off chosen based on empirical data availability), the proportion of people starting ART who were women, and the proportion of people on second line ART. In addition, the model was fitted to data on rates of dropping out of and restarting ART in men and women, and 12-month retention on ART.

Six parameters controlled HIV transmission probabilities in the model, with an additional parameter determining the mean duration of primary infection. One parameter, *baseline_transmission*, determined the mean per sex act transmission probability for a person with a CD4 count between 200 and 350 cells/μl (unweighted average of male to female and female to male transmission probabilities). All other transmission probabilities in the model were calculated relative to this. No limits were placed on the value of *baseline_transmission*, to allow the model to be simultaneously fitted to the sexual behaviour and HIV prevalence data. A second parameter controlled the ratio of male to female and female to male transmission probabilities. The plausible range for this parameter was set to 1.1–4.8, in line with empirical data [14]. As viral loads are correlated with CD4 counts, transmission probabilities are likely to be lower at higher CD4 counts (for post-primary infections), and higher at lower CD4 counts. The plausible range for relative transmission probabilites for people with CD4 counts <200 cells/μl and ≥350 cells/μl were considered to be 1.5–6.7 and 0.42–0.95 respectively [7]. Transmission probabilities may be much higher for people with primary infections. A two-dimensional joint plausible range was placed on input parameters determining the mean duration of primary infection, and the relative transmission probability during primary infection, based on analysis of empirical data by Bellan et al. [15].

Transmission probabilities for people on established ART in the model varied according to their level of drug resistance to the regimen that they were receiving (first or second line ART). With the maximum number of active drugs, the plausible range for relative transmission probabilities was assumed to be 0.04–0.21 [16]. With no active drugs, the probability of transmission was assumed to be the same as it would be if they were not on ART. With intermediate numbers of active drugs, transmission probabilities increased exponentially as the number of active drugs declined.

The model was fitted to the empirical data using history matching with model emulation, a calibration method for complex models which iteratively removes areas of the input space where fits to the data are unlikely to be found [17, 18]. Overall, 96 input parameters were varied during the fitting process, and the model was fitted to 51 outputs. A total of 100 model fits, all consistent with empirical data, were generated using history matching. This approach allowed us to comprehensively incorporate a large number of the potential sources of uncertainty in our results, allowing realistic estimates of uncertainty in model results to be obtained. Full details are given in McCreesh et al. [10].

### Scenarios

A total of nine ART scale-up scenarios were simulated from 2016, making different assumptions about how ART will be scaled-up in Uganda:

1. Baseline. No changes to ART policy or implementation after 2014.
2. Increased HIV testing. The rate of HIV testing was doubled.
3. No CD4 threshold. The CD4 threshold for ART initiation was removed.
4. Improved retention on ART. The rate of dropping out of ART was halved.
5. Increased ART restart rates. The rate of restarting ART after dropping out was doubled.
6. Improved pre-ART care. The rate of dropping out of pre-ART care was halved, the probability of linking to care following a positive HIV test was doubled, and the rate of starting ART from pre-ART (when eligible) was doubled.
7. Improved linkage to care. The probability of linking to care following a positive HIV test was doubled.
8. Universal test and treat (UTT). Combines increased HIV testing rates, no CD4 threshold, and improved linkage to care.
9. Universal test, treat and keep (UTTK). Combines increased HIV testing rates, no CD4 threshold, improved linkage to care, improved retention on ART, and increased ART restart rates.

All changes were implemented from 2016, and all scenarios were run until 2030. A total of 100 model fits were used in the analysis. Results were averaged over 2000 (stochastic) repetitions for each scenario and model fit.

## Results

### Fit to data

**Fig. 1:**
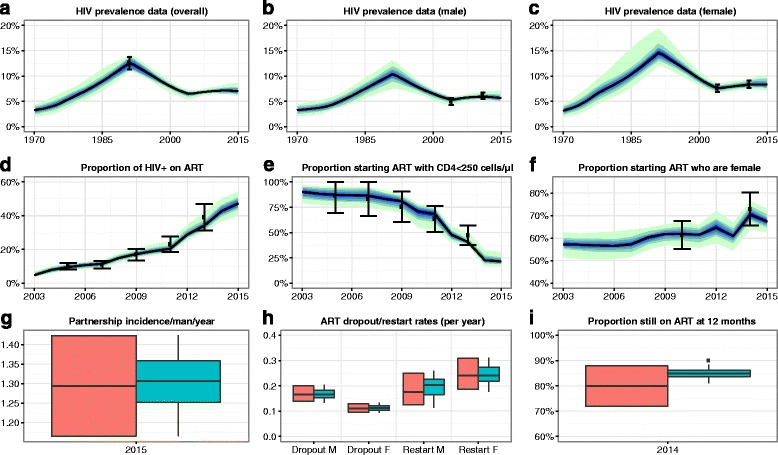
Model baseline fit to empirical data. Graphs **a**-**f**: *Black dots* show the empirical estimates, and the *error bars* show the acceptable ranges for the outputs used in fitting the model. *Black lines* show the median model output. *Blue/green bands* show 10% quantiles of model outputs. The *full width of the band* shows the range of the model output. Graphs **g**-**i**: *Orange boxes* show the empirical data and acceptable ranges. *Green boxes* show the model output. Model fits to the remaining 28 outcomes are shown in McCreesh et al. [10]

The model fitted closely to the acceptable ranges from the empirical data for all 51 outputs. Figure 1 shows model fits in a range of years to HIV prevalence, ART coverage, the proportion of people starting ART with CD4 < 250 cells/μl, and the proportion of people starting ART who are female; male partnership incidence in 2015; male and female ART dropout and restart rates; and 12-month retention on ART. Model fits to an additional 28 outputs are given in McCreesh et al. [10].

### Reductions in transmission input parameter ranges

**Fig. 2:**
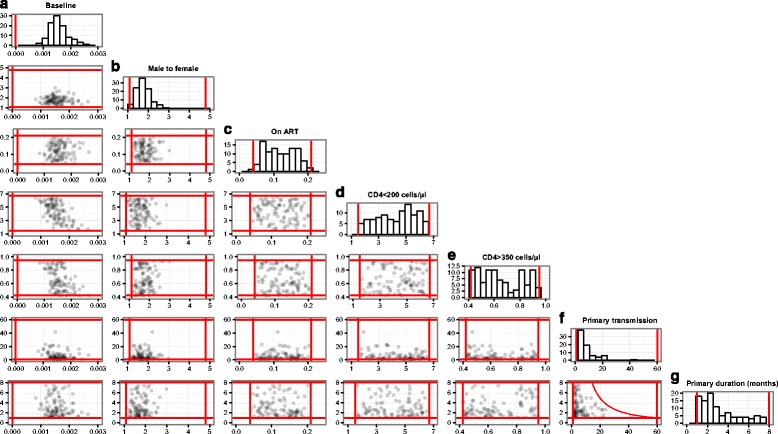
Input parameter initial plausible ranges and fitted values. *Histograms* show the distribution of values in the 100 model fits for the seven transmission input parameters (**a**) baseline transmission; **b**) transmission probability from men to women, relative to transmission probability from women to men; **c**) relative transmission probability on established ART; **d**) relative transmission probability with CD4 count <200 cells/μl; **e**) relative transmission probability with CD4 count >350 cells/μl; **f**) relative transmission probability during primary infection; **g**) duration of primary infection (months)). *Scatter graphs* show the joint distribution of pairs of input parameters. The *red lines* show the initial plausible ranges, before model fitting. All plausible input ranges were independent of the values of other input parameters, with the exception of ‘primary transmission’ and ‘primary duration’ which had a two-dimensional joint plausible range. The plausible upper limit for ‘baseline’ was 1, and is not shown on the graphs

The histograms in Fig. 2 show the distribution of values in the 100 fitted runs for the seven input parameters that control transmission. The red lines show the initial plausible ranges, before model fitting. The ranges of five of the seven transmission input parameters were not reduced during model fitting. In other words, fits were found throughout the whole of the plausible range. The two exceptions were *baseline_transmission* and the ratio of male → female to female → male transmission probabilities, where model fits were limited to within the ranges 0.00083–0.0023 (initial plausible range 0–1) and 1.1–2.7 (initial plausible range 1.1–4.8) respectively.

The scatter graphs in Fig. 2 show the joint distribution of pairs of input parameters. While the distribution of most pairs was uncorrelated, there were negative correlations in the final fitted runs between *baseline_transmission* and the increase in transmission probabilities in people with CD4 counts <250 cells/μl (*r* = −0.63), and between *baseline_transmission* and the decrease in transmission probabilities in people with CD4 counts >350 cells/μl (*r* = −0.44).

### HIV prevalence and ART coverage

**Fig. 3:**
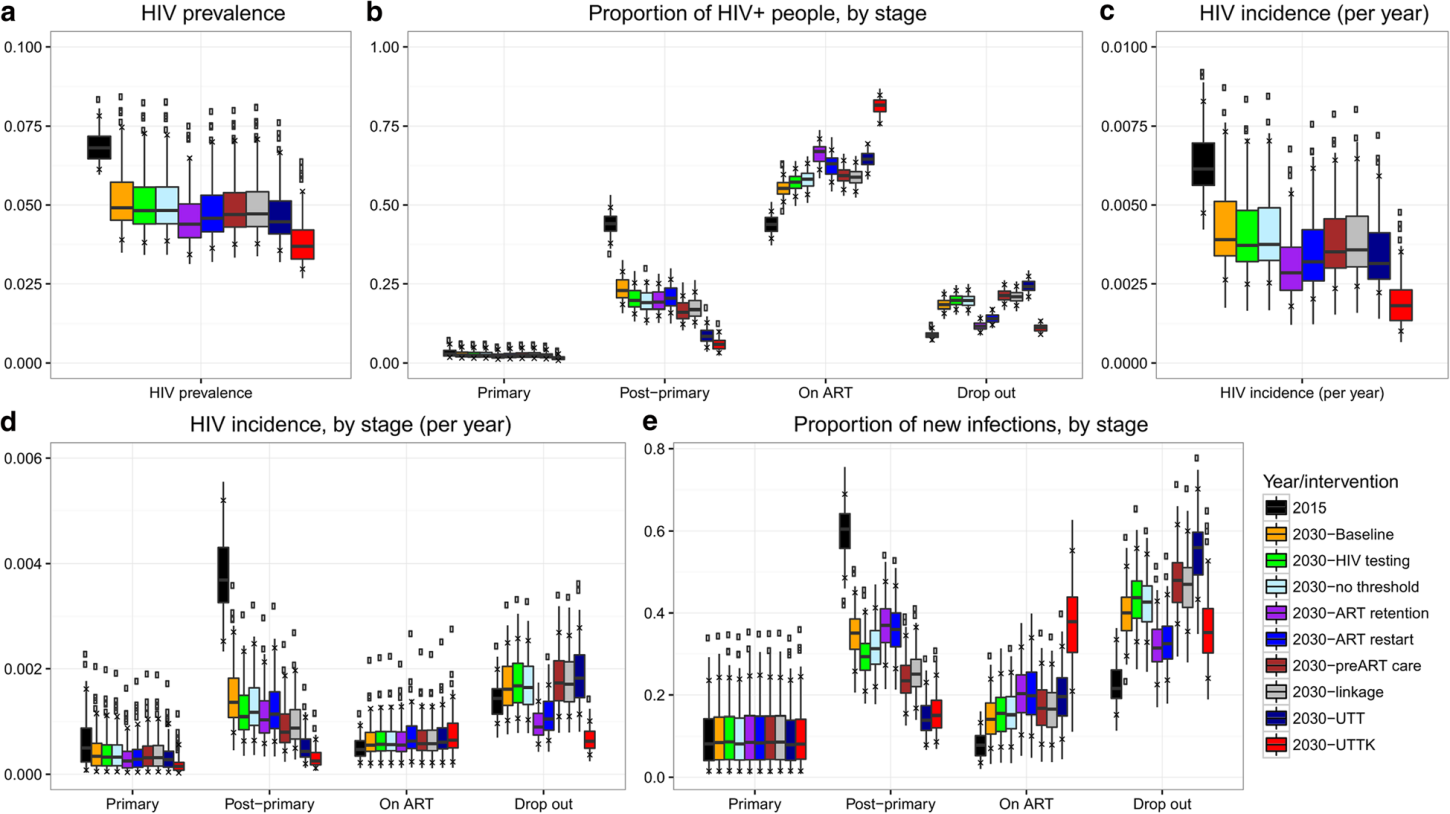
**a**) Overall HIV prevalence in 2015 and 2030 by intervention; **b**) Proportion of HIV+ people by stage; **c**) Overall HIV incidence in 2015 and 2030 by intervention; **d**) HIV incidence by stage in 2015 and by stage and intervention in 2030; **e**) Proportion of new infections due to transmission by people in each stage in 2015 and by stage and intervention in 2030. *Boxes* show the median and 25–75% quartiles. *Whiskers* show the highest/lowest value that is within 1.5 times the inter-quartile range from the 75%/25% quartile. *Crosses* show the 90% plausible range. Results for 2015 are shown in *black*

The projected HIV prevalence in 2030 in adults aged between 15 and 49 years ranged between 4.9% (median; 90% plausible range 3.9%–7.5%) in the baseline scenario to 3.7% (3.0%–5.4%) in the universal test, treat, and keep (UTTK) scenario (Fig. 3a). The projected proportion of HIV positive people who were ART-naïve in 2030 ranged from 26% (21%–32%) in the baseline scenario to 7.6% (5.0%–12%) in the UTTK scenario, with <6% of HIV positive people having primary stage infections in all scenarios and model fits (Fig. 3b). ART coverage of all HIV positive people in 2030 ranged from 55% (51%–60%) in the baseline scenario, up to 82% (76%–85%) in the UTTK scenario. Finally, the proportion of HIV positive people who had dropped out of ART ranged from 11% (9.1%–13%) in the UTTK scenario to 24% (21%–27%) in the universal test and treat (UTT) scenario.

**Fig. 4:**
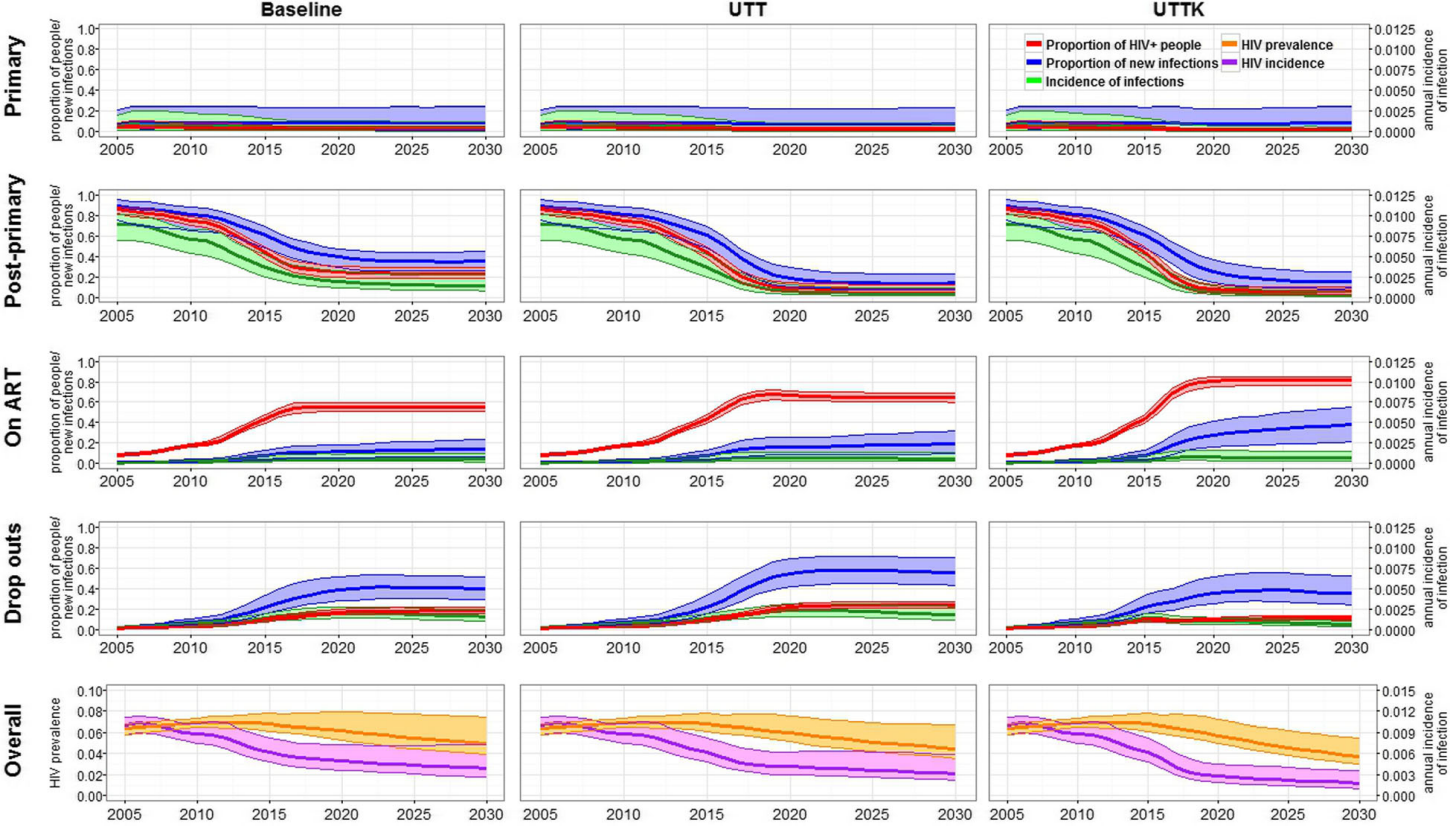
Incidence and proportion of HIV infection due to transmission by HIV+ people in each stage stage (*first four rows*), and overall HIV prevalence and incidence (*bottom row*), for the baseline, universal test and treat (UTT), and universal test, treat, and keep (UTTK) interventions. *Lines* show median, *bands* show 90% plausible range. Note different axis scales for overall

Figure 4 shows trends over time in population composition between 2005 and 2030 in the baseline, UTT, and UTTK scenarios.

### HIV incidence

The overall annual incidence of HIV infection in 2015 in the model was 0.61% (90% plausible range: 0.47–0.83%) (Fig. 3c). Incidence fell between 2015 and 2030, falling to 0.39% (0.26–0.73%) in 2030 in the baseline scenario, and 0.18% (0.10%–0.35%) in the UTTK scenario. In all scenarios, the biggest reductions in incidence occurred as a result of lower transmission from people with post-primary, ART-naïve infections, with the annual incidence of infection by this group falling from 0.37% (0.025%–0.52%) in 2015 to 0.14% (0.079%–0.27%) in 2030 in the baseline scenario and 0.025% (0.0012%–0.066%) in 2030 in the UTTK scenario (Fig. 3d). In all scenarios, the incidence of infection caused by transmission from people with primary infections fell between 2015 and 2030, and the incidence of infection from people on ART increased slightly. Finally, the incidence of infection due to transmission by people who had dropped out of ART fell in scenarios that included interventions to reduce loss to follow up (improved ART retention, increased ART restart rates, and UTTK), and increased in all other scenarios.

### Proportion of HIV incidence

The proportion of new infections resulting from transmission by people with primary infections was very similar in 2015 and in all scenarios in 2030, with the median proportion of transmission ranging from 7.9%–8.6% (Fig. 3e). There was a large amount of uncertainty in the proportion of transmission by people with primary stage infections, with a 90% plausible range of 1.5%–25%.

In 2015, ART-naïve people were responsible for the majority of new infections (70%, 90% plausible range 61%–79%), with people on ART, and ART dropouts, contributing 7.8% (3.5%–13%) and 22% (15%–33%) of new infections respectively. In all scenarios, the proportion of transmission by people with post-primary, ART-naïve infections fell greatly between 2015 and 2030, and the proportion of transmission by people on ART and people who have dropped out of ART increased.

The relative importance of transmission by people with post-primary ART-naïve infections, people on ART, and people who had dropped out of ART in 2030 varied greatly between scenarios. People with post-primary ART-naïve infections were responsible for between 14% (UTT, 7.9%–23%) and 37% (improved retention, 27%–48%) of new infections, people on ART were responsible for between 14% (baseline, 7.0%–24%) and 38% (UTTK, 21%–55%) of new infections, and ART drop-outs were responsible for between 31% (improved retention, 23%–43%) and 56% (UTT, 43%–70%) of new infections.

## Discussion

Model results suggest that, as ART coverage increases in Uganda, people on ART and people who have dropped out of ART will be responsible for an increasingly large proportion of all HIV transmission. In 2015, we estimate that these two groups were responsible for 7.8% (3.5%–13%) and 22% (15%–33%) of all transmission occurring during heterosexual sex respectively. With no change in ART policy or implementation, this may increase to 14% (7.0%–24%) and 40% (29–51%) respectively by 2030. With a universal test and treat (UTT) policy, the proportions may increase to 20% (9.3%–32%) and 56% (43%–70%), and with a (successfully implemented) universal test, treat, and keep (UTTK) policy, the proportions may increase to 38% (21–55%) and 35% (24%–53%).

Our findings have important implications for HIV control. As ART coverage increases in Uganda and elsewhere in sub-Saharan Africa, people who are currently or previously on ART are likely to play an increasingly large role in overall HIV transmission. For this reason, the focus of ‘treatment as prevention’ will increasingly need to shift from finding treatment-naïve HIV positive people and starting them on ART, to also improving treatment adherence and retention on ART, and ensuring that people are promptly switched to 2nd line ART when necessary. Changes in ART regimens and scale-up of viral load testing may also have an important and increasing role in reducing HIV transmission.

The relative contribution of different stages of the ART care pathway to overall transmission will vary by country, depending on details of the country’s ART programme. In general, at higher ART coverages, a higher proportion of transmission will be by people on ART. At the same ART coverage, the proportion of transmission by ART drop-outs will be higher when retention is lower. Estimated ART coverage of all HIV+ adults in Uganda in 2015 was 57% [2]. This is fairly typical for East and Southern Africa, where overall coverage is estimated to be 54%. Estimated coverage varies greatly by country however, ranging from 29% in Angola to 78% in Botswana, and overall coverage is lower in West and Central Africa, at 28%. Representative data on ART retention in sub-Saharan African countries is sparser; however, estimates available from East and Southern Africa range from 66% retention at 12 months in Mozambique, to 93% in Zambia. In Uganda, 12-month retention in 2015 was estimated to be 78%. The proportion of transmission by people on ART and ART drop-outs will therefore vary by country. Nevertheless, these groups are likely to be responsible for an increasing proportion of transmission as ART programmes are scaled-up across sub-Saharan Africa.

In our model, being on ART (with no resistance to the drug regimen) reduced HIV transmission probabilities by 79–96%. This plausible range was equal to the 95% confidence interval from an empirical study of transmission in discordant couples in sub-Saharan Africa [7]. The point estimate from this study was a 92% reduction in transmission probabilities. A second study estimated that ART reduced transmission probabilities by 96% (95% CI: 73–99%) [19]. These reductions are slightly higher than the mean reduction of 88% in our 100 model runs. Adherence is likely to be higher in research study cohorts than in the general population however [20], and therefore our lower simulated mean reduction may be more realistic for Uganda as a whole.

A number of mathematical modelling studies have estimated the proportion of transmission that occurs during primary infection in a wide range of different populations and settings, with estimates ranging from <1% to 93% [4, 21]. Our median estimate of 8.1% of transmission occurring during primary infection in 2015 is low compared to the majority of studies. This is most likely because HIV incidence was declining in 2015 in our model, whereas most previous models have simulated increasing or stable epidemics. All else being equal, when incidence is falling, a lower proportion of HIV positive people will have primary infections, and they will therefore contribute less to overall incidence. Our 90% plausible range was wide (1.5% to 24%), reflecting the large amount of uncertainty that still exists in the duration and relative infectiousness of primary infection. It also demonstrates the importance of fully incorporating levels of uncertainty in input parameters into model projections, something that is often neglected in infectious disease modelling.

## Conclusions

People who are currently or previously on ART are likely to play an increasingly large role in overall transmission as ART coverage increases in Uganda and other sub-Saharan African countries. Improving adherence and retention on ART, and ensuring that people on ART are on effective drug regimens, will be key in reducing the overall incidence of HIV in Uganda. In other words, achieving the 2nd and 3rd UNAIDS ‘90s’ (90% of all people with diagnosed HIV infection receiving sustained antiretroviral therapy by 2020, and 90% of all people receiving ART achieving viral suppression) [22] is likely to become increasingly important to reducing HIV incidence.

## Abbreviations

ART: Anti-retroviral therapy
HIV: Human immunodeficiency virus
UNAIDS: Joint United Nations Programme on HIV/AIDS
UTT: Universal test and treat
UTTK: Universal test, treat, and keep

## References

[1] UNAIDS (2015). UNAIDS strategy 2016–2021.

[2] AIDSInfo [http://www.unaids.org/en/dataanalysis/datatools/aidsinfo].

[3] Uganda AIDS Commission: HIV and AIDS Uganda Country progress report; 2014. Kampala: Uganda AIDS Commission 2015.

[4] Miller WC, Rosenberg NE, Rutstein SE, Powers KA (2010). The role of acute and early HIV infection in the sexual transmission of HIV. Curr Opin HIV AIDS.

[5] Punyacharoensin N, Edmunds WJ, De Angelis D, Delpech V, Hart G, Elford J, Brown A, Gill N, White RG (2015). Modelling the HIV epidemic among MSM in the United Kingdom: quantifying the contributions to HIV transmission to better inform prevention initiatives. AIDS.

[6] Skarbinski J, Rosenberg E, Paz-Bailey G, Hall HI, Rose CE, Viall AH, Fagan JL, Lansky A, Mermin JH (2015). Human immunodeficiency virus transmission at each step of the care continuum in the United States. JAMA Intern Med.

[7] Donnell D, Baeten JM, Kiarie J, Thomas KK, Stevens W, Cohen CR, McIntyre J, Lingappa JR, Celum C, Team PiPHHTS (2010). Heterosexual HIV-1 transmission after initiation of antiretroviral therapy: a prospective cohort analysis. Lancet.

[8] Marzel A, Shilaih M, Yang W-L, Böni J, Yerly S, Klimkait T, Aubert V, Braun DL, Calmy A, Furrer H (2016). HIV-1 transmission during recent infection and during treatment interruptions as major drivers of new infections in the Swiss HIV cohort study. Clin Infect Dis.

[9] Wilensky U (1999). NetLogo.

[10] McCreesh N, Andrianakis I, Nsubuga R, Strong M, Vernon I, McKinley T, Oakley J, Goldstein M, Hayes R, White R. Universal, test, treat, and keep: improving ART retention is key in cost-effective HIV care and control in Uganda. BMC Infect Dis. in press. 10.1186/s12879-017-2420-y PMC5415795 28468605

[11] Kaleebu P, Kamali A, Seeley J, Elliott A, Katongole-Mbidde E. The Medical Research Council (UK)/Uganda virus research institute Uganda research unit on AIDS–‘25 years of research through partnerships’. Tropical Med Int Health. 2014;20(2):E1-E10. 10.1111/tmi.12415 PMC4529486 25354929

[12] McCreesh N, O'Brien K, Nsubuga RN, Shafer LA, Bakker R, Seeley J, Hayes RJ, White RG (2012). Exploring the potential impact of a reduction in partnership concurrency on HIV incidence in rural Uganda: a modeling study. Sex Transm Dis.

[13] Asiki G, Murphy G, Nakiyingi-Miiro J, Seeley J, Nsubuga RN, Karabarinde A, Waswa L, Biraro S, Kasamba I, Pomilla C: The general population cohort in rural south-western Uganda: a platform for communicable and non-communicable disease studies. Int J Epidemiol. 2013:dys234. 10.1093/ije/dys234 PMC3600628 23364209

[14] Nicolosi A, Leite MLC, Musicco M, Arid C, Gavazzeni G, Lazzarin A (1994). The efficiency of male-to female and female-to-male sexual transmission of the human immunodeficiency virus: a study of 730 stable couples. Epidemiology.

[15] Bellan SE, Dushoff J, Galvani AP, Meyers LA (2015). Reassessment of HIV-1 acute phase infectivity: accounting for heterogeneity and study design with simulated cohorts. PLoS Med.

[16] Baggaley RF, White RG, Hollingsworth TD, Boily M-C (2013). Heterosexual HIV-1 infectiousness and antiretroviral use: systematic review of prospective studies of discordant couples. Epidemiology.

[17] Andrianakis I, Vernon IR, McCreesh N, McKinley TJ, Oakley JE, Nsubuga RN, Goldstein M, White RG (2015). Bayesian history matching of complex infectious disease models using emulation: a tutorial and a case study on HIV in Uganda. PLoS Comput Biol.

[18] Andrianakis I, Vernon I, McCreesh N, McKinley TJ, Oakley JE, Nsubuga R, Goldstein M, White RG: Efficient history matching of a high dimensional individual based HIV transmission model. Journal on Uncertainty Quantification in press.

[19] Cohen MS, Chen YQ, McCauley M, Gamble T, Hosseinipour MC, Kumarasamy N, Hakim JG, Kumwenda J, Grinsztejn B, Pilotto JHS (2011). Prevention of HIV-1 infection with early antiretroviral therapy. N Engl J Med.

[20] Daar ES, Corado K (2016). Condomless sex with virologically suppressed HIV-infected individuals: how safe is it?. JAMA.

[21] Suthar AB, Granich RM, Kato M, Nsanzimana S, Montaner JS, Williams BG: Programmatic implications of acute and early HIV infection. J Infect Dis*.* 2015:jiv430. 10.1093/infdis/jiv430 26310309

[22] Joint United Nations Programme on HIV/AIDS: 90–90-90: an ambitious treatment target to help end the AIDS epidemic. Geneva: UNAIDS 2014.

